# COVID-19 Outbreaks in Correctional Facilities with Work-Release Programs — Idaho, July–November 2020

**DOI:** 10.15585/mmwr.mm7016a3

**Published:** 2021-04-23

**Authors:** Eileen M. Dunne, Ellie Morgan, Bruce Wells-Moore, Samuel Pierson, Sandra Zakroff, Lindsay Haskell, Kimberly Link, Jodie Powell, Ian Holland, Kai Elgethun, Christopher Ball, Rene Haugen, Christine G. Hahn, Kris K. Carter, Christine Starr

**Affiliations:** ^1^Epidemic Intelligence Service, CDC; ^2^Idaho Department of Health and Welfare; ^3^Idaho Department of Correction; ^4^Corizon Health; Brentwood, Tennessee; ^5^Central District Health, Boise, Idaho; ^6^Eastern Idaho Public Health, Idaho Falls, Idaho; ^7^Southwest District Health, Caldwell, Idaho; ^8^Agency for Toxic Substances and Disease Registry, Atlanta, Georgia; ^9^Boise VA Medical Center, Boise, Idaho; ^10^Center for Preparedness and Response, CDC.

As of April 16, 2021, U.S. correctional and detention facilities reported 399,631 cases of COVID-19 in incarcerated persons, resulting in 2,574 deaths (*1*). During July 14–November 30, 2020, COVID-19 was diagnosed in 382 persons incarcerated in Idaho correctional facilities with work-release programs. Work-release programs (which place incarcerated persons in community businesses) have social and economic benefits, but might put participants at increased risk for bidirectional transmission of SARS-CoV-2, the virus that causes COVID-19. The Idaho Department of Correction (IDOC) operates 13 state-run correctional facilities, including six low-security facilities dedicated to work-release programs. This report describes COVID-19 outbreaks in five IDOC facilities with work-release programs,* provides the mitigation strategies that IDOC implemented, and describes the collaborative public health response. As of November 30, 2020, 382 outbreak-related COVID-19 cases were identified among incarcerated persons in five Idaho correctional facilities with work-release programs; two outbreaks were linked to food processing plants. Mitigation strategies that helped to control outbreaks in IDOC facilities with work-release programs included isolation of persons with COVID-19, identification and quarantine of close contacts, mass testing of incarcerated persons and staff members, and temporary suspension of work-release programs. Implementation of public health recommendations for correctional and detention facilities with work-release programs, including mass testing and identification of high-risk work sites, can help mitigate SARS-CoV-2 outbreaks. Incarcerated persons participating in work-release should be included in COVID-19 vaccination plans.

A COVID-19 case was defined as detection of SARS-CoV-2 by a nucleic acid amplification test collected from a person incarcerated in an IDOC facility during July 14–November 30, 2020.^†^ Cases were reported to the Idaho Department of Health and Welfare (IDHW).^§^ Facility information and work-release assignments were provided by IDOC. Because IDOC facilities lacked space for individual quarantine and isolation, close contacts^¶^ were quarantined in cohorts for 14 days from the date of exposure. COVID-19 patients were isolated in cohorts or transferred to an IDOC COVID-19 unit** for at least 14 days. Clinical care was provided by a privately held prison health care contractor,^††^ which maintained a COVID-19 log to track testing, symptoms, quarantine, and medical isolation, and regularly shared the data with IDHW. Routine periodic mass testing of staff members and incarcerated persons for SARS-CoV-2 was conducted by IDOC.^§§^ This activity was reviewed by CDC and was conducted consistent with applicable federal law and CDC policy.^¶¶^

During July 14–November 30, 2020, COVID-19 outbreaks occurred at five IDOC facilities with work- release programs. The facilities included four metropolitan community reentry centers (CRCs) with approximately 120 work sites in multiple industries (including manufacturing, food processing, agriculture, construction, retail, and hospitality) and a rural work camp with work sites in the agricultural sector. IDOC provided transportation to and from work sites. A total of 382 COVID-19 cases were identified among incarcerated persons, including 76 (20%) cases in one facility housing women only, and 306 (80%) cases in four facilities housing men only. The median patient age was 37 years (range = 21–69 years). Among COVID-19 patients, 218 (57.1%) were non-Hispanic White persons, 40 (10.5%) were Hispanic or Latino persons, 10 (2.6%) were Black persons, and nine (2.4%) were American Indian or Alaska Native persons; race/ethnicity data were missing for 105 (27.5%) patients. No hospitalizations or deaths occurred.

IDOC facilities provided various housing arrangements for 108–276 persons; the number of COVID-19 cases at each facility ranged from nine to 148 (Table). The total number of incarcerated persons was unavailable because facility populations fluctuated over time, and race and ethnicity data for all incarcerated persons at these facilities were not available. Most cases (64.1%) were identified through mass testing; 13.6% cases were in persons with COVID-19–compatible symptoms. Initial cases at IDOC facilities were identified during July–August 2020, at the same time increases in community incidence occurred in the counties where facilities were located (Figure).

**TABLE:** Correctional facility housing, COVID-19 mass testing results, and COVID-19 cases (N = 382) among incarcerated persons at five correctional facilities with work-release programs — Idaho, July 14–November 30, 2020

Characteristic	Correctional facility					Total (%)
	CRC A	CRC B	CRC C	CRC D	Work camp	
**Capacity no., sex**	115, male	108, male	148, female	113, male	276, male	—*
**Housing style/persons per room**	Four persons per room	Two dorms of 44 and 64 persons	10–12 persons per room	Four persons per room	Seven dorms of 28–60 persons	—
**First case reporting date**	Jul 14	Jul 25	Jul 30	Aug 10	Jul 13	—
**Mass testing date 1**	Aug 4	Aug 13	Aug 4	Aug 31–Sep 3	Jul 27	—
No. of positive/no. tested (%)	38 of 59 (64)	1 of 57 (1.8)	40 of 65 (62)	60 of 79 (76)	11 of 211 (5.2)	150 of 471 (31.8)
**Mass testing date 2**	Nov 9	Nov 9	Nov 9	Sep 29–Oct 1	Sep 29	—
No. of positive/no. tested (%)	1 of 102 (1)^†^	2 of 81 (2.5)	2 of 107 (1.9)	2 of 17 (12)	40 of 191 (20.9)	46 of 498 (9.4)
**Mass testing date 3**	—**^§^**	—	—	—	Oct 16–19	—
No. of positive/no. tested (%)	—**^§^**	—	—	—	49 of 53 (92)	49 of 53 (92)
**Cases detected, by reason for testing (% of cases per facility)**						
Symptomatic^¶^	2 (2.7)	0 (—)	11 (14.5)	3 (4.1)	36 (24.3)	52 (13.6)
Close contact	33 (44.0)	1 (11.1)	17 (22.4)	2 (2.7)	3 (2.0)	56 (14.7)
Mass testing	38 (50.7)	3 (33.3)	42 (55.3)	62 (83.8)	100 (67.6)	245 (64.1)
Prerelease	2 (2.7)	1 (11.1)	2 (2.6)	3 (4.1)	0 (—)	8 (2.1)
Unknown	0 (—)	4 (44.4)	4 (5.3)	4 (5.4)	9 (6.1)	21 (5.5)
**Total cases (% of all cases)**	**75 (19.6)**	**9 (2.4)**	**76 (19.9)**	**74 (19.4)**	**148 (38.7)**	**382 (100.0)**

**Abbreviation**: CRC = community reentry center.

* Dashes in the total column indicate that totals were not generated.

^†^ The person who received a positive test result on November 19, 2020, had previously received a positive result on July 24, 2020, and was not considered to have a new case; this positive result was not included in totals.

^§^ Dashes in this row indicate that no third round of mass testing was conducted at these correctional facilities during the outlined time frame.

^¶^ Symptomatic persons had one or more of these COVID-19–compatible signs and symptoms: fever, cough, shortness of breath, chills, fatigue, muscle aches, headache, new loss of taste or smell, sore throat, congestion, runny nose, and nausea or vomiting. https://www.cdc.gov/coronavirus/2019-ncov/symptoms-testing/symptoms.html

**FIGURE:**
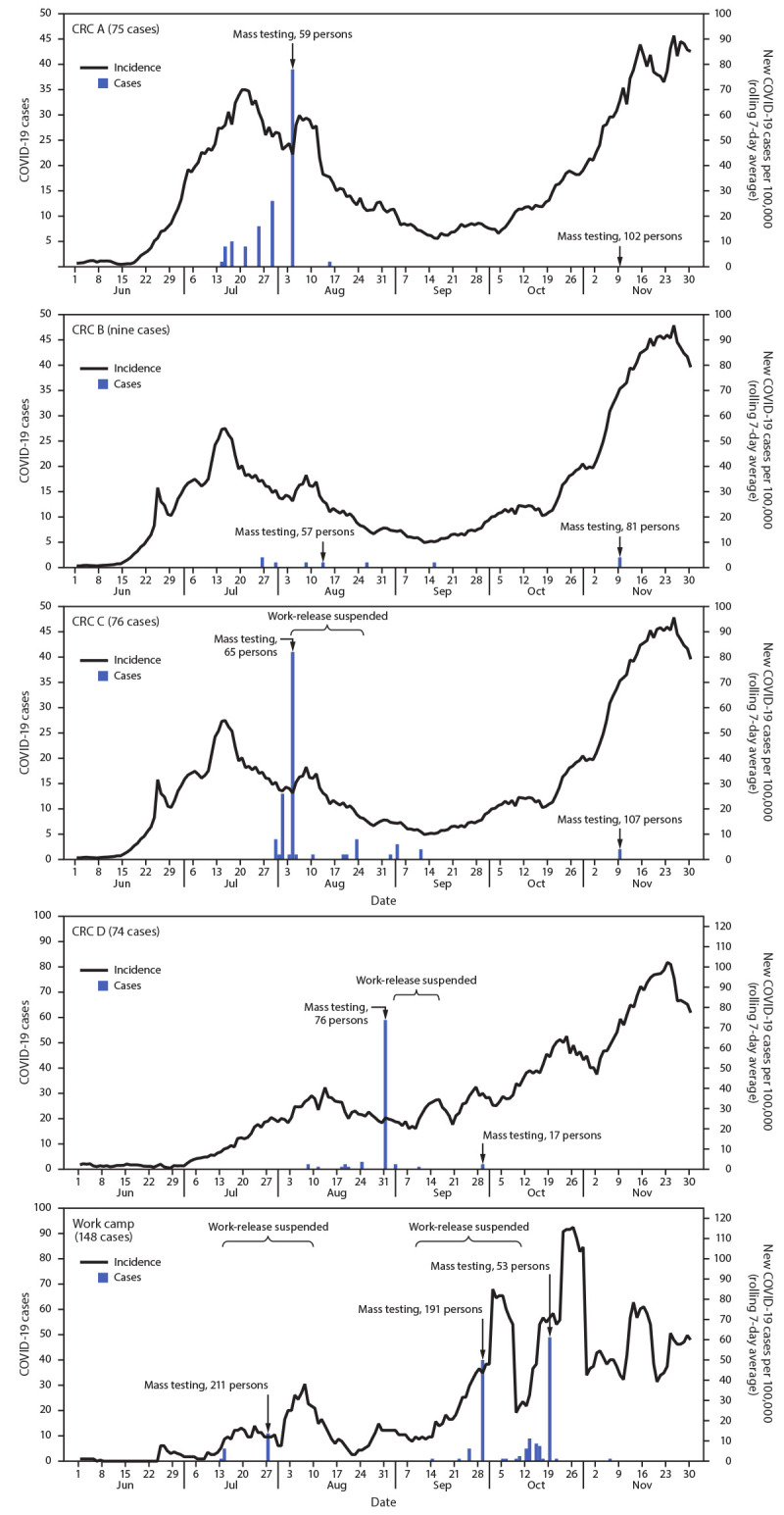
COVID-19 cases among incarcerated persons in four correctional facility community reentry centers (CRCs) and one work camp, by date of specimen collection and county COVID-19 incidence — Idaho, June 1–November 30, 2020

Information on work-release placements was available for CRC A and the work camp. The first COVID-19 case in CRC A was identified on July 14, 2020, in an incarcerated person working at a food processing plant. A COVID-19 outbreak had previously been identified among nonincarcerated employees at this workplace; IDOC was not aware of the ongoing outbreak until notified by public health officials on July 22, 2020, which prompted ongoing communication among IDOC and public health partners. Subsequent IDHW guidance recommended that correctional facilities require work-release sites to notify them of COVID-19 cases among employees and suspend work-release during COVID-19 outbreaks until all close contacts were quarantined and tested.*** At CRC A, cases occurred in 75 incarcerated persons, 16 (21%) of whom worked onsite and 59 (79%) of whom worked at businesses in the community (including 12 persons at the aforementioned food processing plant, five at a car dealership, four at a different food processing plant, four at a manufacturing facility, and 34 at 25 other businesses). After mass testing at CRC A on August 4, 2020, and subsequent isolation of patients and quarantine of close contacts, only one new case was identified at this facility. Seventeen COVID-19 cases were identified at the work camp in July among incarcerated persons working at a single food processing plant. The first two of these cases experienced symptom onset on July 13, 2020, preceded by two cases in nonincarcerated food plant employees with symptom onset on July 9 and July 12, suggesting that the work camp outbreak might have resulted after incarcerated persons were exposed to infection at the work site.

COVID-19 mitigation measures at all 13 IDOC-operated correctional facilities included intensified cleaning and mandatory use of face masks for staff members and incarcerated persons (hand soap and four reusable face masks distributed to each incarcerated person), and periodic SARS-CoV-2 mass testing. Universal temperature checks and symptom screenings were conducted daily and at entry. New admissions were quarantined for 14 days and tested for SARS-CoV-2 before transfer to general housing. The percentage of positive test results from mass testing at IDOC facilities with work-release ranged from 1% to 92% (Table). All cases identified during mass testing occurred in persons who were asymptomatic at the time of testing.

Mitigation strategies at IDOC facilities with work-release programs included 1) providing temperature checks and symptom screening before incarcerated persons departed to work sites and upon their return; 2) ensuring that face masks were worn during transport; 3) requiring employers to provide a COVID-19 safety plan; 4) documenting work-site safety measures, including physical distancing, mask use, and hand hygiene; and 5) conducting employer site checks to confirm safety standards were being maintained. Three IDOC facilities with work-release programs erected temporary housing structures to create more space for isolation and quarantine. Work-release was suspended temporarily at three facilities (CRC C, CRC D, and the work camp) to help control outbreaks (Figure).

Collaborative public health response initiatives were also implemented. IDHW hosted weekly calls with representatives from IDOC, the health care contractor, local public health districts, and Boise VA Medical Center laboratory to share information on cases, clinical capacity, mass testing, and public health guidance. IDOC regularly provided lists of CRC work sites to IDHW; public health officials notified IDOC of work sites considered to be high-risk for COVID-19 transmission (e.g., congregate setting without mitigation measures) or those experiencing active outbreaks. These collaborations increased testing availability and prompted IDOC to reassign some work-release participants to lower-risk work sites.

## Discussion

CDC COVID-19 guidance advises correctional facilities to consider suspending work-release programs, especially when the work-release assignment is in a congregate setting, such as a food processing plant (*2*). COVID-19 outbreaks at two state correctional facilities described in this report were linked to work-release at food processing plants. Epidemiologic evidence suggests that these plants were the likely source of the outbreaks. COVID-19 outbreaks with no known links to food processing plants occurred at three other IDOC facilities operating work-release programs and at four of five IDOC facilities without external work programs (Idaho Department of Correction, unpublished data, 2020). These findings indicate that incarcerated persons at correctional facilities that operate work-release programs might be at risk for acquiring SARS-CoV-2 during placement, in addition to the risks they face in a congregate housing setting.

Prompt isolation of persons with COVID-19, quarantine of close contacts, and mass testing helped control SARS-CoV-2 transmission in IDOC correctional facilities operating work-release programs. Mass testing of incarcerated persons detected more cases than did symptom-based or close contact testing. Most COVID-19 cases occurred in asymptomatic persons, providing further evidence that symptom screening alone is insufficient for case detection (*3*–*5*). The absence of hospitalizations and deaths might reflect differences in participants in work-release programs compared with the overall IDOC prison population, in which five COVID-19–related deaths and 18 hospitalizations occurred through November 30, 2020 (Corizon Health, Inc., unpublished data, 2020).

Challenges for outbreak control in correctional facilities with work-release programs are similar to those usually faced by correctional and detention facilities, including congregate living and lack of available housing to quarantine close contacts individually (*6*). However, facilities with work-release programs have the added risk for SARS-CoV-2 transmission from the work setting, particularly when work placements are in congregate facilities such as food processing plants. Incarcerated persons might be disinclined to report symptoms because of reluctance to isolate or other reasons (*7*). In addition, some correctional facilities might not be able to implement certain CDC-recommended mitigation measures, such as distribution of alcohol-based hand sanitizers and maintaining full-time medical staff members and medical isolation.

The findings in this report are subject to at least six limitations. First, information on individual work assignments of COVID-19 patients was only available for two correctional facilities. Second, it is unknown whether SARS-CoV-2 transmission from incarcerated workers to nonincarcerated employees occurred. Third, COVID-19 mitigation measures in the surrounding communities were not assessed. Fourth, testing practices might have varied across facilities, and mass testing was not conducted at set intervals. Fifth, self-reporting of symptoms was considered unreliable, and the presence of symptoms before or after testing was not recorded. Finally, findings are not generalizable to all correctional and detention facilities with work-release programs.

The benefits of work-release programs include the increased likelihood of postrelease employment and decreased recidivism (*8*). However, work-release might lead to exposure of incarcerated persons to SARS-CoV-2 at work sites in the community and subsequent introduction into the correctional facility environment. Correctional and detention facilities, public health officials, and work sites should collaborate to ensure that incarcerated persons participating in work-release are included in COVID-19 vaccination plans and scheduled clinics. Measures to reduce SARS-CoV-2 transmission, including mass testing for early detection of SARS-CoV-2 and collaboration with public health officials to identify work sites with higher risk for SARS-CoV-2 transmission, should be considered for correctional and detention facilities operating work-release programs.

**Summary**

**What is already known about this topic?**

Correctional and detention facilities face unique challenges for controlling transmission of SARS-CoV-2. Work-release programs, which place incarcerated persons in community businesses, might pose additional risks.

**What is added by this report?**

As of November 30, 2020, a total of 382 outbreak-related COVID-19 cases were identified among incarcerated persons at five Idaho correctional facilities with work-release programs; two outbreaks were linked to work at food processing plants.

**What are the implications for public health practice?**

Correctional facilities operating work-release programs should implement measures to reduce SARS-CoV-2 transmission, including mass testing and working with public health officials to identify high-risk work sites. Incarcerated persons participating in work-release should be included in COVID-19 vaccination plans.

## Footnotes

* One of the five facilities operates vocational work projects.

† Reverse transcription–polymerase chain reaction tests were done at the Idaho Bureau of Laboratories or Boise VA Medical Center (TaqPath COVID-19 Combo Kit, Thermo Fisher Scientific Inc.), or at IDOC (Abbott ID NOW COVID-19, Abbott Diagnostics Scarborough, Inc.) during July 14–November 30, 2020.

§ SARS-CoV-2 test results were reported to IDHW in accordance with Coronavirus Aid, Relief, and Economic Security Act requirements (https://www.cdc.gov/coronavirus/2019-ncov/lab/reporting-lab-data.html) and Idaho reportable disease rules (https://healthandwelfare.idaho.gov/providers/reportable-diseases/idaho-reportable-diseases).

¶ A close contact was defined as a person who shared a sleeping area, worked at the same work-release site, was transported in the same vehicle, or was within 6 ft of a person infected with SARS-CoV-2 for a total of ≥15 minutes during a 24-hour period (beginning 2 days before illness onset for symptomatic close contacts, and 2 days before test specimen collection for asymptomatic close contacts) until the time the patient was isolated. Close contacts were tested when they developed one or more symptoms compatible with COVID-19; in some circumstances asymptomatic close contacts were tested as part of outbreak response or a mass testing event. https://www.cdc.gov/coronavirus/2019-ncov/php/contact-tracing/contact-tracing-plan/appendix.html#contact

** A dedicated space in an IDOC correctional facility for medical isolation and monitoring of persons who received a diagnosis of COVID-19.

†† Corizon Health, Inc., Brentwood, Tennessee, http://www.corizonhealth.com

§§ IDOC conducted SARS-CoV-2 mass testing of staff members and incarcerated persons across all 13 facilities. Mass testing events were conducted multiple times per week, with schedules and locations determined by IDOC; priority was given to new outbreaks and units housing older adults (≥60 years of age) or persons with medical conditions associated with increased risk for severe illness from SARS-CoV-2. https://www.cdc.gov/coronavirus/2019-ncov/need-extra-precautions/people-with-medical-conditions.html

¶¶ 45 C.F.R. part 46, 21 C.F.R. part 56; 42 U.S.C. Sect. 241(d); 5 U.S.C. Sect. 552a; 44 U.S.C. Sect. 3501 et seq.

***
https://coronavirus.idaho.gov/wp-content/uploads/2020/08/082120_COVID-19-guidance_work-release-from-correctional-facilities_20200821.pdf
